# Identification of weak and gender specific effects in a short 3 weeks intervention study using barley and oat mixed linkage β-glucan dietary supplements: a human fecal metabolome study by GC-MS

**DOI:** 10.1007/s11306-017-1247-2

**Published:** 2017-08-18

**Authors:** Alessia Trimigno, Bekzod Khakimov, Josue Leonardo Castro Mejia, Mette Skau Mikkelsen, Mette Kristensen, Birthe Møller Jespersen, Søren Balling Engelsen

**Affiliations:** 1 Department of Food Science, Faculty of Science, University of Copenhagen, Rolighedsvej 26, 1958 Frederiksberg C, Denmark; 2 Department of Agricultural and Food Sciences, Alma Mater Studiorum - University of Bologna, Piazza Goidanich 60, 47521 Cesena (FC), Italy; 3 Department of Nutrition, Exercise, and Sports, Faculty of Science, University of Copenhagen, Rolighedsvej 26, 1958 Frederiksberg C, Denmark

**Keywords:** Mixed linkage β-glucan, Short chain fatty acids, Fecal metabolome (GC-MS), Chemometrics, ASCA

## Abstract

**Introduction:**

Mixed-linkage (1→3),(1→4)-β-d-glucans (BG) reduce cholesterol level and insulin response in humans. Despite this, their role in human metabolism and a mode of action remains largely unknown.

**Objectives:**

To investigate the effects of three structurally different BG on human fecal metabolome in a full cross-over intervention using GC-MS metabolomics.

**Methods:**

Over three weeks of intervention, young healthy adults received food supplemented with BG from oat, two different BG from barley or a non-fiber control in a full cross-over design. Untargeted metabolomics and short chain fatty acid analysis was performed on day three fecal samples. ANOVA-simultaneous component analysis was applied to partition the data variation according to the study design, and PLS-DA was used to select most discriminative metabolite markers.

**Results:**

Univariate and multivariate data analysis revealed a dominating effect of inter-individual variances followed by a gender effect. Weak effects of BG intake were identified including an increased level of gamma-amino-butyrate and palmitoleic acid in males and a decreased level of enterolactone in females. Barley and oat derived BG were found to influence the human fecal metabolome differently. Barley BG increased the relative level of formate in males and isobutyrate, isovalerate, 2-methylbutyrate in females. In total 15, 3 and 11 human fecal metabolites were significantly different between control vs. BG, control vs. oat BG, and barley BG vs. oat BG, respectively.

**Conclusions:**

The study show that human fecal metabolome largely reflects individual (∼28% variation) and gender (∼15% variation) differences, whereas the treatment effect of the BG (∼8% variation) only manifests in a few key metabolites (primarily by the metabolites: d-2-aminobutyric acid, palmitoleic acid, linoleic acid and 11-eicosenoic acid).

**Electronic supplementary material:**

The online version of this article (doi:10.1007/s11306-017-1247-2) contains supplementary material, which is available to authorized users.

## Introduction

Mixed-linkage (1→3),(1→4)-β-d-Glucans (BG) have been investigated in in vivo and in vitro studies, for their functional properties and health beneficial effects (Chen and Raymond 2008; Daou and Zhang 2012). BG are plant cell-wall polysaccharides and have been proven to exert beneficial effects in the gastro-intestinal tract (Cloetens et al. 2012; Lam and Cheung 2013) due to their physicochemical properties in aqueous environment such as thickening and gelling agent (Izydorczyk and Biliaderis 2000). The reported bioactivities of BG include: (1) lowering of the post-prandial blood insulin and glucose levels (Battilana et al. 2001; Jenkins et al. 2002; Frank et al. 2004; Biorklund et al. 2005; Beck et al. 2009a), (2) decrease of the cholesterol level in hypocholestorolaemic humans as well an in animals (Kalra and Jood 2000; Kerckhoffs et al. 2002; Delaney et al. 2003; Biörklund et al. 2005; Queenan et al. 2007), (3) reduction of blood pressure (Keenan et al. 2002; Liatis et al. 2009) and (4) increased cholecystokinin production (Bourdon et al. 1999; Beck et al. 2009a). It has been hypothesized that the ingestion of large amount of BG can only induce modest alteration in human blood (Battilana et al. 2001).

Despite the rich literature about the health beneficial effects of BG, a number of studies have not been able to show significant effects of BG. Frank et al. (Frank et al. 2004), investigated the effects of two oat-derived BG on blood lipids, glucose, insulin and tocopherol where BG showed no effect. Queenan et al. (2007) studied concentrated oat BG and observed that this soluble dietary fiber did not influence blood glucose and insulin concentration, weight, homocysteine and C-Reactive protein. Ibrügger et al. (2013) showed that BG do not lower a cholesterol level in young healthy adults after 3-weeks of BG consumption (3.3 g/day), and in a follow-up study Mikkelsen et al. (Mikkelsen et al. 2014) showed no systematic metabolic differences between BG and control group using NMR metabolomics of blood plasma.

While the reasons for many no-effect studies may be multi-facetted, including lack of power and the use of inappropriate study design, the health beneficial potential of BG still calls for debate and the mechanisms are still under investigation. The health beneficial effects of BG have been related to their unique molecular block structures (Wood 2007; Sullivan et al. 2013). The difference between the barley and oat BG relies primarily in their block ratio of cellotriosyl and cellotetraosyl units connected by β-d-(1→3) linkages (Mikkelsen et al. 2013). This ratio is generally lower in oat than in barley and it is considered as an important molecular parameter of the BG (Hughes et al. 2008). This variation also depends on the specific plant genotype (Mikkelsen et al. 2013) and growing conditions (Herrera et al. 2016). Oat BG have a higher water solubility than barley BG (Mikkelsen et al. 2013) and it is thus possible that BG from different sources with various structures and physicochemical properties may have a different impact on the metabolism of the host. Moreover, it has not been studied yet whether the effects of BG depend on the phenotype of the host.

This study investigates the same human intervention study as described in Ibrügger et al. (2013) and Mikkelsen et al. (2014). The study included three BG derived from oat, mother-line barley (BOMI) and a mutant barley (*lys5.f*) which were provided as supplements to low-fat yogurt or blackcurrant-syrup beverage and the same product was investigated without the addition of any BG as a control. While the previous two studies were focused on the blood metabolome, this study investigates effects of the BG on the fecal metabolome. It is hypothesized that the human fecal metabolome which is less influenced by homeostasis, may exhibit significant excretion effects related to the BG supplement intake. The fecal metabolome has until recently not been scrutinized in great detail, but it represents a direct reflection of the crucial function of the gut and its microbiota to human health (Xu et al. 2014). Dietary fibers such as BG are known to be fermented by the microbiota into SCFA, which can be used by the mammalian host to produce the aforementioned beneficial effects. It is thus of fundamental interest to investigate whether the fecal metabolome is affected by the BG intake, and if so, how it is modulated by the different BG preparations. This study employs untargeted GC-MS metabolomics as well as GC-MS analysis of short-chain fatty acids (SCFA) along with advanced multivariate data analysis in order to study possible effects of BG on the fecal metabolome in humans.

## Materials and methods

### Experimental design

**Fig. 1:**
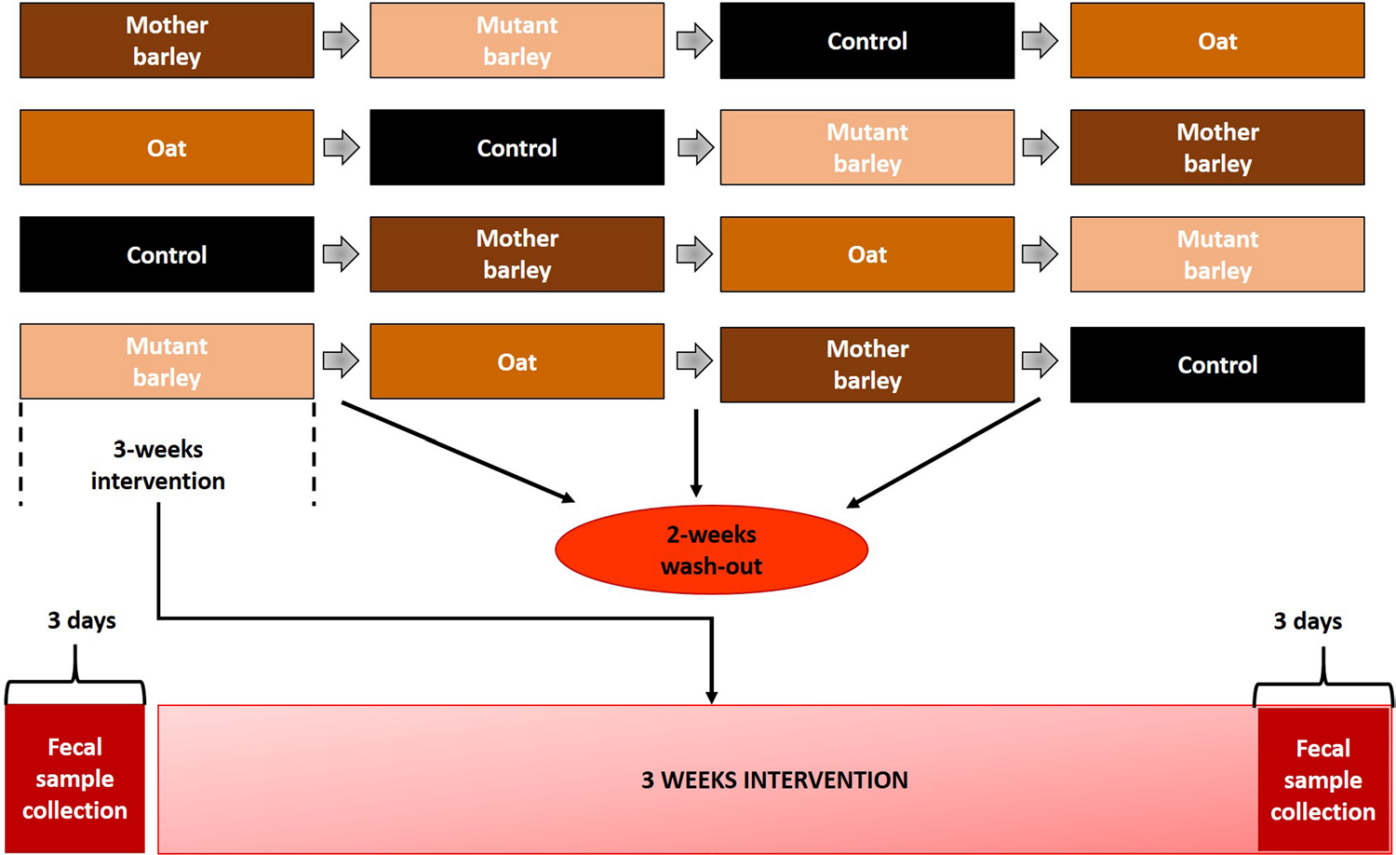
Design of experiment. The trial is a randomized full cross-over, where the subjects undergo 3-weeks interventions of each treatment, followed by a 2-weeks wash-out period. Fecal samples are collected during the 3 days period before the treatment started and during the last 3 days period of each intervention. The treatments consisted of: mother barley BG (**a**), oat BG (**b**), control (**c**) and mutant barley BG (**d**)

The study design was single-blinded, randomized and 4-armed cross-over and each treatment lasted 3 weeks and had at least 2 weeks of wash-out period (Fig. 1). The 16 recruited subjects were normo-cholesterolaemic and followed their normal dietary habits apart from a few restrictions (Ibrügger et al. 2013). The study was designed based on a power calculation (Kristensen et al. 2011, 2012) in order to assess total cholesterol differences with sufficient power and allowing a 10% drop-out rate. Fourteen subjects completed the study (eight females and six males). The experimental design is described in Fig. 1.

The oat BG were purified from a concentrate (Promoat™; Biovelop, Kimstad, Sweden) and barley BG were obtained either from a mother line BOMI (6% BG) or a mutant line *lys5.f* (16.5–19.8% BG) (Munck et al. 2004). The isolation of BG from grains was obtained by hot water and enzymatic hydrolysis, employing a modified procedure from Kvist and Lawther (Kvist and Lawther 2011), as described by Mikkelsen et al. (2013). The pre-hydrated BG preparations were given to the participants as a beverage with blackcurrant syrup (Minimum^®^) or as a low-fat vanilla yoghurt (Arla Cheasy^®^), with 1.65 g of BG per preparation, thus allowing the subjects to take the recommended daily dose of at least 3 g of BG (21 CFR 101.81, 62 FR 3584, January 23 1997; EFSA Journal 2010).

### Metadata collection

The diet of each participant was recorded for 4 days and entered into the Dankost 3000 dietary assessment software (Dankost 3000, version 2.5; Danish Catering Center), in order to calculate the mean global intake of energy and macronutrients (carbohydrates, fats, proteins and alcohol) and fibers. Twenty-four blood parameters, including fasting concentrations of total, HDL and LDL cholesterol, plasma glucose and triacylglycerides were measured as reported by Mikkelsen et al. (2014).

Fecal samples were collected during 3 days, both before and after intervention (Fig. 1). Fecal samples of each participant collected during the 3 days period were pooled and energy was measured using a bomb calorimeter (Ika-calorimeter system C4000; Heitersheim). One sample from a participant, collected in one out of 3 days period, was used for pH measurement (PH-208; Lutron Electonic Enterprise) after being mixed with demineralized water (1:1) and homogenized. Further description of samples collection and analysis are reported elsewhere (Ibrügger et al. 2013; Mikkelsen et al. 2014). The final metadata variables consisted of blood parameters, anthropometric measurements [height, weight, age, body mass index (BMI)], dietary information (levels of hunger, satiety, fullness) and fecal parameters (e.g. pH, energy, weight, frequency).

### GC-MS analysis

Untargeted GC-TOF-MS metabolomics of fecal slurry was performed on 1 ml fecal slurry which was mixed with 1 ml of a buffer followed by freeze dying and methanol extraction. Following a two step derivatization, methoximation and trimethylsilylation, the samples were analyzed using a HT Pegasus GC-TOF-MS system (LECO Corporation, Saint Joseph, USA). Targeted analysis of fecal SCFAs was performed using 0.5 ml of fecal slurry, which was mixed with 1 ml of 0.3 M oxalic acid and analyzed by GC-MS (Agilent Technologies, California, USA). The Detailed description of the GC-MS analysis is given in the Supplementary Material.

### Data analysis

The data generated from the untargeted GC-TOF-MS metabolomics, targeted SCFA analysis, and metadata variables were analyzed individually as well as combined using univariate, including ANOVA, and multivariate, principal component analysis (PCA) (Hotelling 1933) and partial least squares-discriminant analysis (PLS-DA) (Ståhle and Wold 1987), data analysis methods. According to the experimental design (Fig. 1), several factors including BG treatment (TR), gender (**GN**), and individual variations (**IN**), and their two and higher order interactions may contribute to a significant variation. Subsequently, these variations may confound or interact with BG related metabolic changes. Therefore, ANOVA-simultaneous component analysis (ASCA) (Smilde et al. 2005) was performed for partitioning variations in the data into several effect matrices (e.g. **X**_**TR**_, **X**_**GN**_, **X**_**IN**_) using the study design. Subsequently each effect matrix was further evaluated using PCA and PLS-DA as described in the previous study (Khakimov et al. 2016). PLS-DA based classification models, using variable selection, were developed for identification of metabolite markers related to the BG treatment, using delta metabolomics data, **ΔX** = **X**_**T1**_ − **X**_**T0**_, where **X**_**T0**_ is the measurement before the intervention started and **X**_**T1**_ is the measurement after the intervention. The GC-MS data were autoscaled before multivariate data analysis which was performed using Matlab (R2014b The Mathworks Inc.). Details about ANOVA and ASCA calculations are reported in the Supplementary Materials.

## Results and discussion

The untargeted GC-TOF-MS metabolomics data included 279 variables that covered metabolite classes such as organic acids, carbohydrates, amino acids, and fatty acids. A total of 164 out of 279 variables were identified at level 2, according to the Metabolomics Standard Initiative (MSI) (Sumner et al. 2007). The SCFA data consisted of eight variables including acetic acid, formic acid, propionic acid, isobutyric acid, butyric acid, isovaleric acid, 2-methylbutyric acid, valeric acid and one unknown compound. A total of 44 metadata variables were used in this study. Table S1 in Supplementary Material contains a list of all the variables investigated in this study. The final size of the data was 105 samples and 331 variables.

**Table 1:** Major discriminant variables reflecting BG treatment effects. The arrow indicates which of the two treatments showed an increase in relative levels of a marker variable

Untargeted GC-TOF-MS variables		
Metabolite/Variable	Comparison	P-value
Males		
Unknown 16	Control vs. Oat ↑	0.00550
1,2-Propanediol-2TMS	Control ↑ vs. BG	0.0399
d-2-Aminobutyric acid-2-TMS	Control vs. BG ↑	0.0225
Palmitoleic acid-1TMS	Control vs. BG ↑	0.0335
Linoleic acid-1TMS	Control ↑ vs. BG	0.0381
11-Eicosenoic acid-1TMS	Control ↑ vs. BG	0.0314
Urea-2TMS	Barley ↑ vs. Oat	0.0465
Unknown 59 (sugar)	Barley vs. Oat ↑	0.0213
Females		
Enterolactone	Control ↑ vs. Oat	0.0179
Unknown 82	Control ↑ vs. Oat	0.0132
Hexamethyldisilthiane	Control ↑ vs. BG	0.00555
Ethylene glycol-2TMS	Control vs. BG ↑	0.0491
Mannose, 6-deoxy-2,3,4,5-tetrakis-*O*-(trimethylsilyl)-, L-	Control ↑ vs. BG	0.0123
Unknown 72	Control ↑ vs. BG	0.0236
4-*O*-β-Galactopyranosyl-d-mannopyranose, octakis(trimethylsilyl) ether (isomer 2)	Control ↑ vs. BG	0.0058
Unknown 79	Control ↑ vs. BG	0.0373
Enterolactone	Control ↑ vs. BG	0.0028
Tetracosanoic acid-1TMS	Control ↑ vs. BG	0.0434
Unknown 82	Control ↑ vs. BG	0.0009
Tocopherol-γ-tms-derivative	Control ↑ vs. BG	0.0024
Unknown 89	Control ↑ vs. BG	0.0042
Glycolic acid-2TMS	Barley ↑ vs. Oat	0.0077
3-Hydroxybutanoic acid-2TMS	Barley ↑ vs. Oat	0.0345
Unknown 7	Barley ↑ vs. Oat	0.0160
Unknown 37	Barley ↑ vs. Oat	0.0398
Myo-Inositol-5TMS, bis(trimethylsilyl) phosphate	Barley ↑ vs. Oat	0.0250

SCFA and metadata variables		
Males		
Formic Acid	Barley ↑ vs. Oat	0.0099
Total plasma SCFA	Barley ↑ vs. Oat	0.0324
Subjective fullness	Barley vs. Oat ↑	0.0172
Females		
Isobutyrate	Barley ↑ vs. Oat	0.0108
IsoValerate	Barley ↑ vs. Oat	0.0290
2MethylButyrate	Barley ↑ vs. Oat	0.0205

Initial PCA analysis of the **ΔX** and **X**_**T1**_, using all three datasets separately as well as combined dataset, showed no clear separation of individuals based on the BG treatment, but individual and gender related variations were more pronounced (Fig. S1 in Supplementary Material). In order to study the effects related to the study design, three main effects, **TR, GN** and **IN**, and their two-factor interaction effects were studied by ASCA using the **ΔX** and **X**_**T1**_ datasets. Variations derived from the study design were decomposed as following:

1 $${\mathbf{X = X}}_{{{\mathbf{MEAN}}}} {\mathbf{ + X}}_{{{\mathbf{IN}}}} {\mathbf{ + X}}_{{{\mathbf{TR}}}} {\mathbf{ + X}}_{{{\mathbf{RESIDUALS}}}}$$

2 $${\mathbf{X = X}}_{{{\mathbf{MEAN}}}} {\mathbf{ + X}}_{{{\mathbf{TR}}}} {\mathbf{ + X}}_{{{\mathbf{GN}}}} {\mathbf{ + X}}_{{{\mathbf{TR \times GN}}}} {\mathbf{ + X}}_{{{\mathbf{RESIDUALS}}}}$$

where **X** stands for either **ΔX** or **X**_**T1**_.

Due to the fact that **GN** is nested within **IN**, it is impossible to evaluate all three factors simultaneously. Moreover, this study design does not allow to estimate **IN** and **TR** interaction term due to the consumption of degrees of freedom (Eq. 1). In order to evaluate **TR** and **GN** interaction terms using ASCA, the final dataset was balanced by removing four individuals where measurement time points were missing, which left 36 samples for the analysis. Both, **ΔX** and **X**_**T1**_ datasets were investigated using Equations 1 and 2 using the combined data from GC-TOF-MS, SCFA and metadata (Tables S2 and S3 in Supplementary Material).

Using the combined dataset, **X**_**T1**_ revealed significant (P = 1.8E−13) **IN** effect which explains 27.7% variation, while **TR** effect was not significant (P = 0.73) and explained 6.6% variation. The **IN** effect was no longer significant (P = 0.64, 17.9% variation) when combined data was evaluated using **ΔX**. Likewise, **TR** effect was also not significant (P = 0.78, 7.9% variation). A similar picture emerged when GC-TOF-MS data was evaluated for the **IN** and **TR**, using both **ΔX** and **X**_**T1**_ datasets. The SCFA data depicted large **IN** effect from **X**_**T1**_ (P = 1.49E−10) explaining 56.2% variation, though the **TR** effect remained not significant (P = 0.16, 5.3% variation). However, the **ΔX** of the SCFA data showed that the **TR** effect was on the border of being significant (P = 0.052) and explained the largest **TR** variation, 12.9%, when compared to the other datasets. Similar to the SCFA data, the metadata showed large **IN** effect (P = 1.0E−9, 44.9% variation) from **X**_**T1**_ and a nearly significant **TR** effect (P = 0.063, 10.5% variation) when evaluated by **ΔX**.

The **GN** effect and its interaction with **TR** were evaluated according to the Eq. 2 using both the **ΔX** and **X**_**T1**_ datasets. The **GN** effect was significant from **X**_**T1**_ in all datasets including GC-TOF-MS (P = 0.02), SCFA (P = 2.47E-4), metadata (P = 3.05E−7) and the combined dataset (P = 1.99E-4). The explained variances were 5.2, 22.1, 14.9, 6.9%, respectively. The interaction term between **GN** and **TR** was not found to be significant in any cases.

The initial PCA and ASCA analysis suggests that the major variation on human fecal metabolome as well as on metadata were largely related to inter-individual and gender differences. However, a weak effect of BG treatment was observed in SCFA and metadata using **ΔX**. The sections below explore the **GN** effect in detail using **X**_**T1**_ and the **TR** effect using **ΔX** for the combined dataset. The effects will be further discussed on metabolite levels identified as being discriminant markers between males and females as well as for the different BG treatments.

### Gender effect

**Fig. 2:**
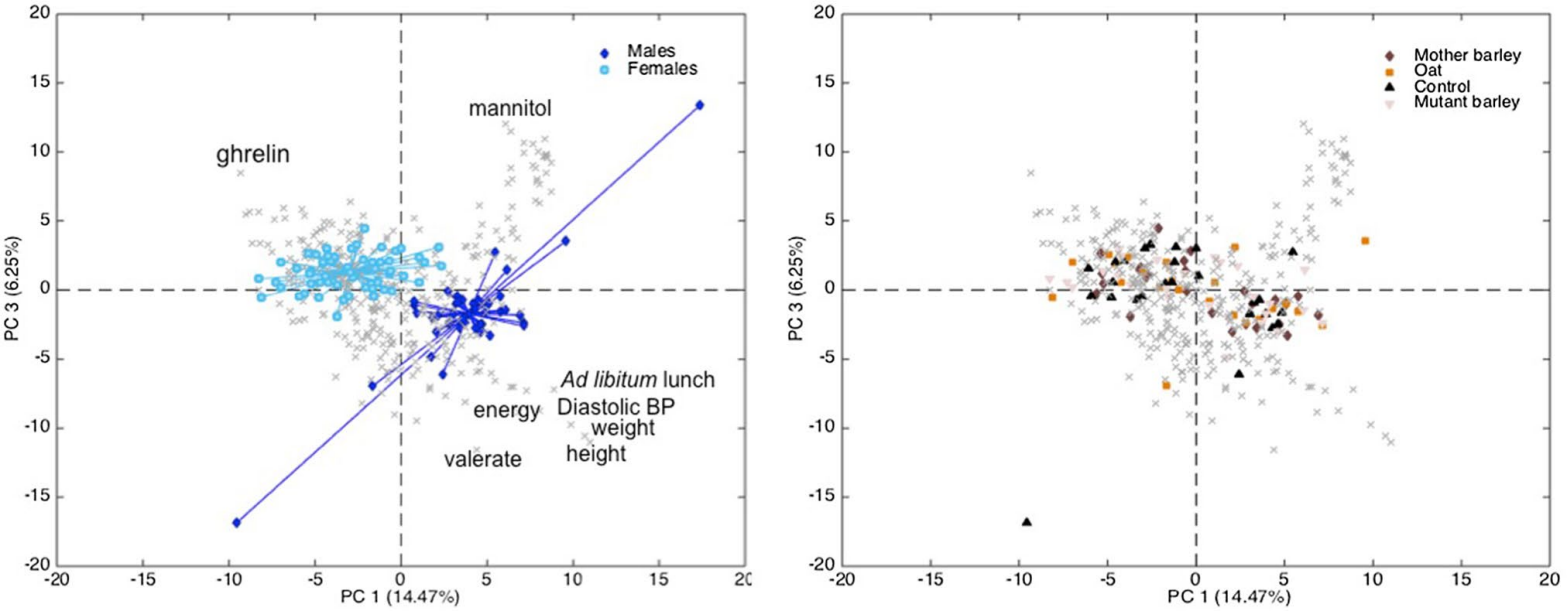
Bi-plot showing PC1 vs. PC3 scores and loadings of the PCA model calculated on the gender effect separated data matrix, **X**_**GN**_. On the left, the colors represent the gender classes, whilst on the right, the same plot is *colored* according to the treatment. PC3 was chosen instead of PC2 due to the fact that it showed better separation between the gender classes

A PCA model of the combined dataset using **X**_**T1**_, showed a gender related clustering amongst the participants (Fig. S1 in Supplementary Material). This effect became even more pronounced on the PCA model developed on the gender effect separated matrix, **X**_**GN**_ Eq. (2) using ASCA (Fig. 2).

In order to find the most discriminative variables separating men from women, PLS-DA based variable selection was performed on the gender separated matrix, **X**_**GN**_, as described previously (Khakimov et al. 2016). PLS-DA models were validated using a double-check validation scheme as described in the literature (Szymanska et al. 2012). The final PLS-DA model, which included one principal component, possessed a high classification power with approximately 19% misclassification rate using independent test set samples (30% of total samples). Identified metabolite markers from the PLS-DA based variable selection were further tested using a one-way ANOVA. Five out of 7 variables selected from the multivariate PLS-DA approach were also found to be significant in a univariate one-way ANOVA and they include diastolic pressure, body weight, energy intake, total ghrelin and ad libitum lunch (Fig. 2).

One of the gender metabolite markers was ghrelin which was higher in females compared to males. Ghrelin is a peptide hormone formed by 28 amino-acids and it is secreted by endocrine cells in the stomach (Abu-Fahra et al. 2014). In a study on 24 non-diabetic adults focused on the effect of different nutrient loads (glucose, protein and lipids) upon ghrelin levels, Greenman et al. (2004) found that ghrelin levels were significantly higher in females both at fasting state and after the glucose and lipid intervention. Gender-related difference in total plasma ghrelin was also reported by Makovey et al. (2007), who measured it in opposite-gender twins, and by Abu-Fahra et al. (2014), who investigated plasma ghrelin levels of 359 residents of Kuwait. The fact that there is significant difference in ghrelin levels between the two genders suggests that this factor could have an impact on the response to the intervention. In fact, ghrelin has been shown to stimulate appetite and release of cortisol, decrease energy metabolism and promote adipogenesis (Makovey et al. 2007). The levels of ghrelin in plasma are increased in fasted states and are lowered after meals (Makovey et al. 2007). It is thus likely that ghrelin has an influence on metabolism and appetite in humans and therefore could cause different behavior and responses to the selected treatment between males and females. Indeed, the administration of ghrelin to mice and rats has been shown to cause an increased food intake and weight gain (Tschöp et al. 2000).

### Treatment-related effects

The initial hypothesis of this study was that the three structurally different BG may exert slightly varying hypocholesterolemic effects in humans. The previous study showed that oat BG had a great functional potential, with a greater decrease in fasting triacylglycerol, which might be due to physicochemical properties such as higher viscosity and solubility (Ibrügger et al. 2013). The consumption of oat and barley derived BG by young healthy adults showed no clear treatment-related effect when the plasma metabolome was investigated by NMR spectroscopy (Mikkelsen et al. 2014). However, the study revealed the presence of subject-dependent lipoprotein plasma profiles and variations due to gender, BMI and diet.

In this study, ASCA and PCA analysis performed on the **ΔX** showed a partial discrimination of the treatments, oat BG subjects were more different than others (data not shown). However, PCA of **ΔX** could not capture an effect of all BG groups. This may be due to a small treatment effect compared to the large inter-individual and gender variation. In order to reduce complexity, three different combinations: oat BG vs. control, oat BG vs. barley BG, and control vs. all types of BG, were studied in detail.

A previous study (Frank et al. 2004) showed that the intake of oat BG lowered cholesterol level in females more than in males, thus suggesting a modulation of the effect of BG intake depending on gender. In this study the effects of BG treatments were not always consistent for both genders (data not shown) and the effects were often more clear when one gender was studied at a time. Below we will describe effects of treatments separately for each gender.

#### Control vs. oat

**Fig. 3:**
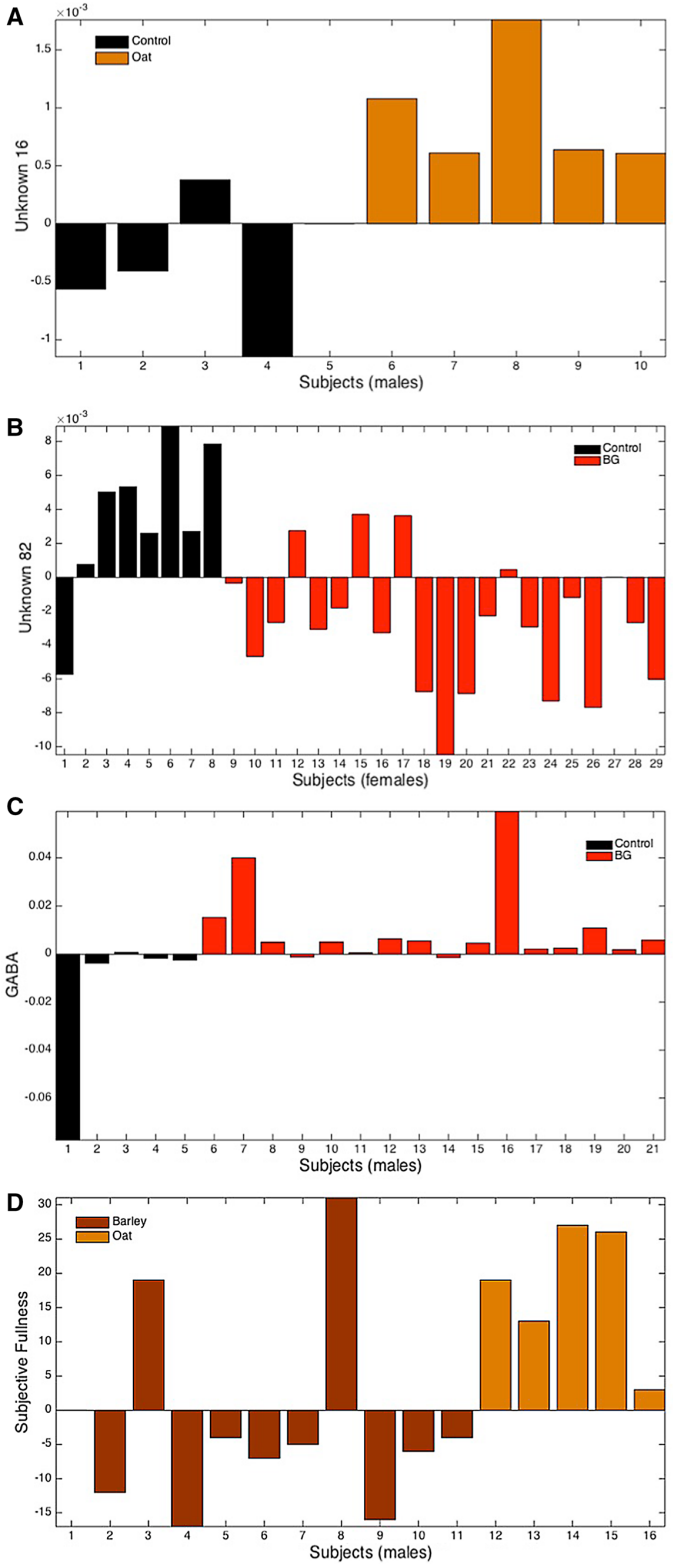
Relative concentrations of major variables found to be discriminative for treatments, using **ΔX** data: **a** unknown 16 for control vs. oat in males; **b** unknown 82 for control vs. BG in females; **c** GABA for control vs. BG in males and **d** subjective fullness for barley vs. oat in males

The effect of the oat BG intake was mostly pronounced among males, where one particular variable, unknown 16, was able to discriminate oat BG group from a control group (Fig. 3). Few other variables were also consistently detected being markers for oat BG group, including females. Two metabolites were mostly responsible for this discrimination and they both decrease during the oat treatment. One of them is enterolactone. Further results are reported in Table 1 and the discussion is found in the Supplementary Material.

#### Control vs. BG

Evaluation of all types of BG treatment groups simultaneously against the control group using PLS-DA and ANOVA revealed 11 variables that were identified as markers for BG treatments in females. Most of the discriminative metabolites decreased during the BG treatment including mannose and unknown 82 (Fig. 3). The decrease of sugars could be ascribed to the delay and decrease in starch metabolism and carbohydrate absorption due to the action of BG (Andrade et al. 2016).

In males, five variables were able to discriminate between the two groups, and among these gamma-amino-butyrate (GABA) was the most powerful marker which was increased in the BG groups (Fig. 3). However, a similar intervention study involving BG intake derived from a whole grain barley pasta have shown contradicting results where GABA level was decreased as a function of BG intake among elderly males and females (De Angelis et al. 2015). Palmitoleic acid level was elevated in the BG groups which is in agreement with a previous study involving oat bran consumption (Gu et al. 2015). Moreover, we found that lipids including linoleic acid, 11-eicosenoic acid and 1,2-propanediol levels were decreased in the BG groups.

#### Barley vs. Oat

**Fig. 4:**
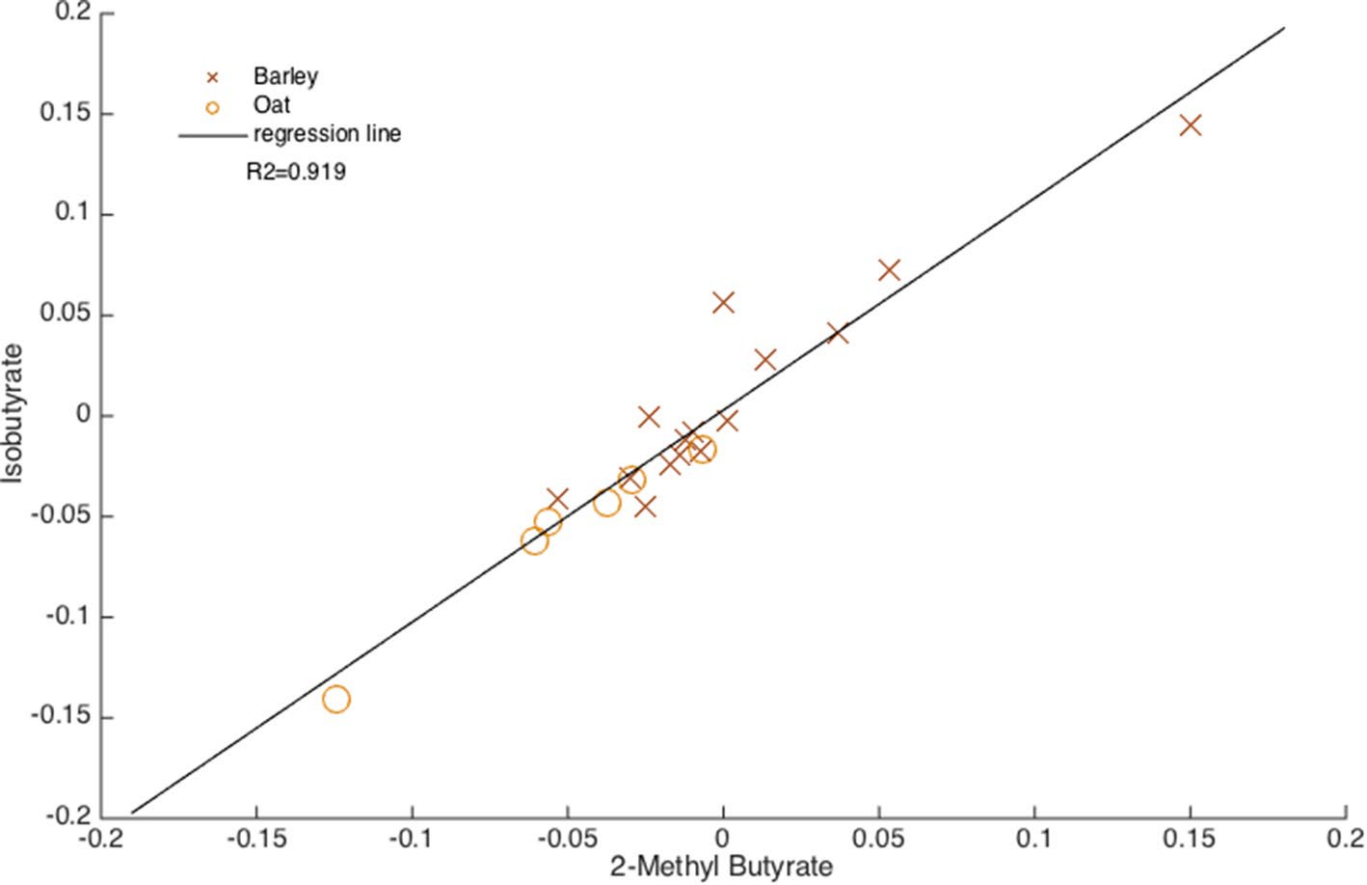
Correlation between 2-methyl butyrate and isobutyrate using ΔX data, differentiating the barley BG and oat BG groups among female subjects

A total of eight variables were found to be different between female subjects who received either barley BG or oat BG. PLS-DA and ANOVA revealed that the levels of glycolic acid, 3-hydroxybutanoic acid and myo-inositol, together with the SCFA including isobutyric acid, isovaleric acid and 2-methylbutyric acid were higher in individuals receiving barley BG (Table 1). A high inter-correlation of some SCFA suggests presence of a strong relation in their metabolism which is high likely to be influenced by the BG consumption (Fig. 4). In fact, the anaerobic intestinal microbiota ferment BG, after these are digested into glucopyranosides, into SCFA (Topping and Clifton 2001). Likewise, in this study, De Angelis et al. (2015) also found increased level of isobutyric acid in fecal samples as a function of BG intake through a pasta meal in a 2-month intervention study involving 26 healthy subjects. In addition, Nilsson et al. (2008) found increased fecal concentrations of isobutyrate and isovalerate after a dietary supplementation of BG enriched oat bran in 25 young healthy individuals.

In males, formic acid, total plasma SCFA and urea were increased during barley BG treatment, whilst the concentration of unknown 59, tentatively identified as sugar metabolite, was decreased. In agreement with this study, Nilsson et al. (2008) reported a decreased level of fecal formic acid after 4 weeks of an oat bran intervention involving 25 young healthy adults. A decrease in formic acid suggests that the level of this metabolite is largely reduced due to the barley BG intake, since it is continuously synthesized in colon during a microbial fermentation. The subjective fullness was found to be higher as a result of the oat BG intervention which is in accordance with previously published studies (Juvonen et al. 2009; Vitaglione et al. 2009) and might be related to the generally higher solubility and viscosity of oat BG (Juvonen et al. 2009). The fact that subjective fullness appears as significant only for males, may be due to the different ghrelin values found between the two genders (see Sect. 3.1), as this hormone is directly related to food intake.

A previous study showed that oat BG improved satiety in both females and males and the actual food intake of a second meal after a BG enriched meal was lower in males (Beck et al. 2009a). Conflicting results, though, are also present in literature: a review by Cloetens and colleagues presents 31 studies on the effects of BG intake and among these 8 showed no significant results in any measured parameters, whilst many others only had partial results (Cloetens et al. 2012). Therefore, a debate around the weight control effect of BG enriched meals are still present since the effect can depend on the food item into which BG are added, the type of BG (Jenkins et al. 2002; Bjorklund et al. 2005) and maybe even this effect can be different in different subject groups.

### Main treatment related results

**Fig. 5:**
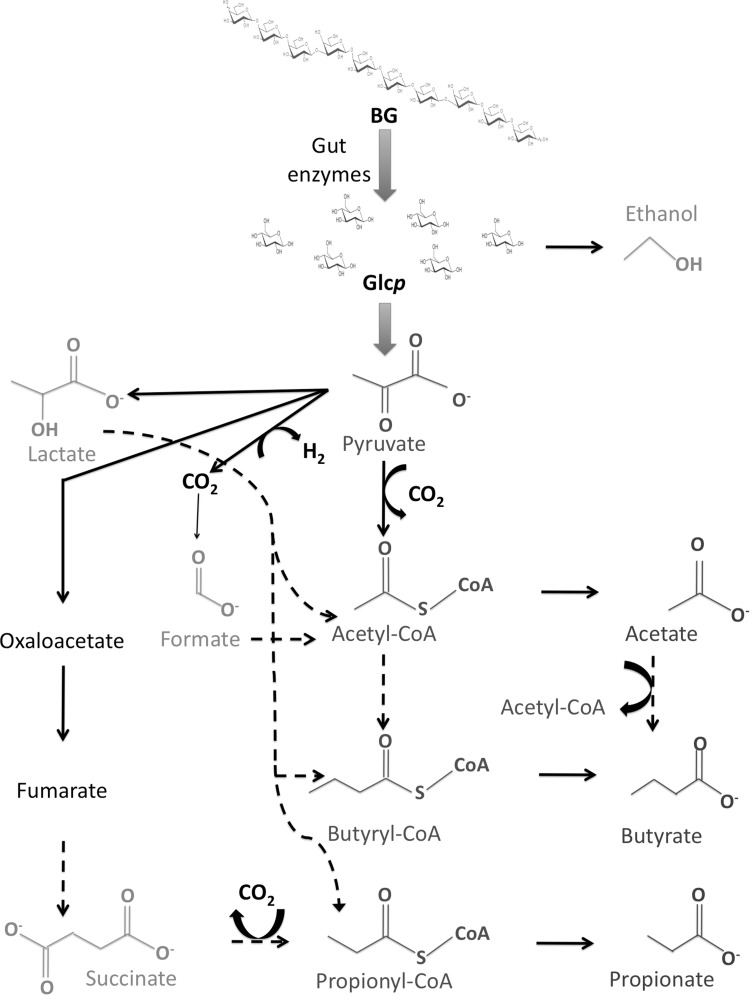
Metabolic destiny of BG through fermentation by the gut microbiota. Glucopyranosides (Glc*p*) are metabolized into pyruvate, which in turn can be metabolized into to various other metabolites including SCFA. Metabolites in *green* are intermediate compounds, whilst metabolites in dark red are end products

This study has revealed relatively weak effects of BG intake when compared against the control group receiving a non-fiber glucagel, although a stronger discrimination was found when the barley BG treatment was compared with the oat BG treatment. Moreover, the SCFA were found to be modulated differently by oat and barley BG which in turn indicate that different BG can alter the anaerobic bacterial fermentation and result in different metabolic profiles. SCFA are directly correlated to BG, since they originate from their metabolism by the gut microbiota (Fig. 5). Formate, which was found to be higher in males after the barley BG intervention, is in fact one of the first product originating from BG gut degradation, deriving from the metabolism of pyruvate. The other SCFA found in different levels between oat and barley BG treatments, derive from later stages of pyruvate metabolism and transformation of butyrate.

The employed oat BG extract was more soluble than the barley counterparts and had a higher viscosity. These two factors of the oat BG makes it more effective towards the improvement of the wellbeing (Cloetens et al. 2012). However, only a weak effect was observed between oat and control treatments. The experienced weak effects can be related to different reasons. The investigated subjects were all young healthy adults with relatively fast metabolism and with no specific cholesterol or diet-related issues and this might be the reason why the intervention did not show specific effects in cholesterolaemia and related anthropometric and blood parameters. In fact, it has been observed before that the cholesterol-lowering property of BG was more pronounced in subjects with initially higher cholesterolaemia (Önning et al. 1999; Frank et al. 2004).

In addition, the number of subjects involved in this study was relatively small and a large part of the data variation was related to the gender and inter-individual differences. Most importantly, the dietary intervention was performed by the addition of only the recommended daily dose of BG and only for a short period of time (3 weeks). Thus, in a group of healthy young people this may not be sufficient to show strong metabolic effects. This is supported by the fact that Nilsson et al. (2008) observed an increase in fecal SCFA only after 8 weeks of intervention on a healthy cohort. They hypothesized that the colonic microbiota is more stable in healthy subjects, thus a change in its composition requires more time. Moreover, they experience a decreased in carboxylic acid concentrations after 12 weeks of intervention, possibly due to an adaptation of the microbiota.

The relative low dose of BG, 3.3 g/day, corresponding to the FDA and EFSA health claim, might also influence the results. In fact, most of the interventions employed higher doses of BG (Mäkeläinen et al. 2007; Beck et al. 2009b; Lyly et al. 2010), especially when added as a supplement to other foods. However, even at higher doses, some studies have showed no effect of BG (Davy et al. 2002; Keogh et al. 2003; Beck et al. 2010). Major metabolic effects due to the BG intake in this study were more clearly manifested in some individuals, which indicate that the effect of BG might have a rather personalized function. Therefore the **IN** effect was found to be the most dominating. For all these reasons, further studies with much larger cohorts are necessary to better investigate the fecal metabolome variation after BG intervention. This should also allow to better estimate phenotypic and individual responses to BG supplements. In addition, a higher dose of BG and/or hypercholesterolemic subjects might be necessary to evaluate effects and the plausible differences between the structurally different BG.

Moreover, the complexity of the metabolomics data and the fact that only a relatively scarce literature exists, especially for human studies and related to specific dietary intervention, made it difficult to assign many metabolites with confidence. The intricate interaction between food-derived molecules, digestion and microbiota, generates a complex pattern of fecal metabolites that is not yet clearly defined, especially in interventions where fibers like BG are used and are fermented by the gut microflora. The gut microbiota is a very intricate and its mechanisms and regulations remain largely unknown. Variations in its composition and behavior can depend on many factors including diet and the ethnicity and genetic make-up of the subjects, which can have an influence on its composition (Escobar et al. 2014; Kwok et al. 2014; Maukonen and Saarela 2015). Therefore, differences between the studied population and the ones considered in previous research might be another reason for the discrepancies found, together with the strong inter-individual effect. Thus, more controlled and carefully design intervention considering all is needed prior to reveal insights into the mode of action and functionality of BG in humans.

## Conclusion

This study demonstrated relatively weak, but consistent metabolic effects in the human fecal metabolome as a function of structurally different BG supplement intake. The weak trends found in this study were well in agreement with the previous findings, which proved only slight tendencies during the intervention and showed that inter-individual and gender-specific effects were more prominent. This underlines the fact that the efficacy of BG interventions depend on factors, such as the length of the intervention, the dose of employed BG supplements, the number of subjects, and perhaps even other phenotypic characteristics. In females, the enterolactone level was decreased when subjects underwent to the BG intake, whilst in males, the GABA level increased after the same treatment. SCFA discriminated oat BG group and barley BG group subjects both for males and females. Barley BG increase specific SCFA levels in both genders, whilst oat BG seems to be more effective in subjective fullness in males. Changes observed in the SCFA profiles as a result of BG intake were in accordance with previously published studies and confirmed the function of in bacterial fermentation in the gut.

In conclusion, this study represents a step forward in the analytical pipeline towards new research with a larger and more diverse cohort and using a much longer intervention-time with time-dynamic fecal sampling. The chemometrics approaches employed here showed that it is necessary to investigate all the possible effects that may impact data, and to validate observed metabolic effects using independent test set samples. It should be emphasized that the present study represents a relative untargeted fecal metabolomics study and that it is possible that a more targeted approach towards, for example, bile acids and/or cholesterol metabolism can provide additional information.

## Electronic supplementary material

Below is the link to the electronic supplementary material.

Supplementary material 1 (DOC 569 KB)
