# Supplementary material for: Identification of weak and gender specific effects in a short 3 weeks intervention study using barley and oat mixed linkage β-glucan dietary supplements: a human fecal metabolome study by GC-MS
Source: Metabolomics. 2017 Aug 18;13(10):108. doi: 10.1007/s11306-017-1247-2 (PMC5562775; doi:10.1007/s11306-017-1247-2)
Supplement: Supplementary file 1 — Supplementary material 1 (DOC 569 KB) [file 11306_2017_1247_MOESM1_ESM.doc]

Identification of weak and gender specific effects in a short 3 weeks intervention study using barley and oat mixed linkage β-glucan dietary supplements: A human fecal metabolome study by GC-MS

Alessia Trimigno^1,2^, Bekzod Khakimov^1^, Josue Leonardo Castro Mejia^1^, Mette Skau Mikkelsen^1^, Mette Kristensen^3^, Birthe Møller Jespersen^1^, Søren Balling Engelsen^1^*

**Affiliations**

^1^Department of Food Science, Faculty of Life Sciences, University of Copenhagen, Rolighedsvej 30, 1958 Frederiksberg C, Denmark

^2^Department of Agricultural and Food Sciences, Alma Mater Studiorum - University of Bologna, Piazza Goidanich 60, 47521 Cesena (FC), Italy

^3^Department of Nutrition, Exercise, and Sports, Faculty of Life Sciences, University of Copenhagen, Rolighedsvej 26, 1958 Frederiksberg C, Denmark

***Corresponding author:** Professor Søren Balling Engelsen

Email: [se@life.ku.dk](mailto:se@life.ku.dk), Mobile: +45 20 20 00 64

**Untargeted GC-TOF-MS metabolomics of fecal slurry**

1 ml fecal slurry (as described above) was mixed with 1 ml of Sterile PBS (5.7 mM Na_2_HPO_4_, 24.3 mM NaH_2_PO_4_, 450 mM NaCl, pH 7.4) and vortexed for 10 s at 3,000 rpm. Samples were frozen using liquid nitrogen and freeze-dried overnight. 20 mg of each freeze-dried sample were mixed with 1 ml of 99.98% methanol containing 10 ppm palmitic acid methyl ester and 10 ppm sorbitol as internal standards. Then samples were vortexed for 10 s at 3,000 rpm and centrifuged for 30 min at 12k g at 4°C. 50 µl of supernatant (corresponding to 1 mg of dried fecal material) was then transferred into 200 µl glass inserts and completely dried using ScanVac (Labogene, Lynge, Denmark) at 40°C and by spinning at 1000 rpm. Immediately after drying, samples were sealed with air tight magnetic lids into 2.0 ml GC-MS vials and derivatized in two steps using a Dual-Rail MultiPurpose Sampler (MPS) (Gerstel, Mülheim an der Ruhr, Germany); 1. Addition of 10 µl of methoximation reagent (20 mg ml^-1^ Methoxiamine hydrochloride in dry pyridine) followed by agitation at 45°C for 90 min by mixing at 750 rpm, 2. Addition of 40 µl of trimethylsilylation reagent, trimethylsilyl cyanide (TMSCN) (Khakimov et al. 2013) followed by agitation at 45°C for 45 min by mixing at 750 rpm. All steps involving sample derivatization and injection were automated using the MPS which was equipped with a sample agitation unit. This procedure insured precise derivatization time and reproducible sample injection. Immediately after derivatization, 1 μl of the derivatized sample was injected into a cooled injection system (CIS4) (Gerstel, Mülheim an der Ruhr, Germany) in splitless mode. The septum purge flow and purge flow to split vent at 2.5 min after injection were set to 25 and 15 ml min^-1^, respectively. Initial temperature of the CIS4 port was 45 °C, and heated at 12 °C s^-1^ to 320 °C (after 30 s of equilibrium time), where it was kept for 10 min. After heating, the CIS4 port was gradually cooled to 250 °C at 5 °C s^-1^, and this temperature was kept constant during the run. The GC-MS setup was made by combining an Agilent 7890B gas chromatograph (GC) (Agilent Technologies, California, USA) with a time-of-flight (TOF) mass spectrometer, HT Pegasus TOF-MS, (LECO Corporation, Saint Joseph, USA). GC separation was performed on a Zebron ZB 5% Phenyl 95% Dimethylpolysiloxane column (30 m with I.D. 250 μm and film thickness 0.25 μm) with a 5 m inactive guard column (Phenomenex, Torrance, USA). A hydrogen generator, Precision Hydrogen Trace 500 (Peak Scientific Instruments Ltd, Inchinnan, UK) was used to supply a carrier gas at the constant column flow rate of 1.0 ml min^-1^. The initial temperature of the GC oven was set to 40 °C and held for 2 min followed by heating at 10 °C min^-1^ to 320 °C and kept for an additional 6 min, making the total run time 36.0 min. Mass spectra was recorded in the range of 45–600 m/z with a scanning frequency of 10 scans sec^-1^, and the MS detector and ion source was switched off during the first 6.3 min of solvent delay time. The transfer line and ion source temperature were set to 280 °C and 250 °C, respectively. The mass spectrometer was tuned according to manufacturer’s recommendation using perfluorotributylamine (PFTBA). The MPS and GC-TOF-MS was controlled using vendor software Maestro (Gerstel, Mülheim an der Ruhr, Germany) and ChromaTOF (LECO Corporation, Saint Joseph, USA). Samples were randomized prior to derivatization and GC–MS analysis. In order to monitor instrument performance, a blank sample containing only derivatization reagent, a control sample (a pooled sample), and an alkane mixture standard sample (all even C10-C40 alkanes at 50 mg L^-1^ in hexane) were injected after every 10 real samples.

The raw GC-TOF-MS data was processed using Statistical Compare toolbox of the ChromaTOF software (Version 4.50.8.0) with following settings; the raw data was used without smoothing prior to peak deconvolution, baseline offset was set to 0.8, expected average peak width was set to 1.5 sec, signal-to-noise was set to ≥10, peak areas were calculated using deconvoluted mass spectra (DT), common *m/z* ions of derivatization products were determined as 73, 75, and 147, and deconvoluted mass spectra were also used for peak identification using LECO-Fiehn and NIST11 library (NIST, Maryland, USA). The library search was set to return top 10 hits with EI-MS match of >75% using normal-forward search and with a mass threshold of 20. Deconvoluted peaks were aligned across all samples using following settings; retention time shift allowance of <2 sec, EI-MS match of >90%, mass threshold of >20, and present in >80% of all pooled control samples.

**Targeted fecal short-chain fatty acid analysis**

SCFA were analyzed using GC-MS from 0.5 ml of fecal slurry, which was mixed with 1 ml of 0.3M oxalic acid containing 10 ppm 2-ethyl-butyrate as internal standard. Samples were vortexed for 1 min, centrifuged at 20 °C for 20 min at 12,000 g. followed by filtration using a 0.45 µm centrifugal filter (Millipore UFC30HV00) and an obtained aliquot was used for GC-MS analysis. The GC-MS consisted of an Agilent 7890A GC and an Agilent 5973 series MSD. GC separation was performed on a Phenomenex Zebron ZB-WAXplus column (30 m × 250 μm × 0.25 μm). A sample volume of 1 μl was injected into a split/splitless inlet at 285 °C using split mode at 2:1 split ratio. Septum purge flow and split flow were set to 13 ml min^-1^ and 2 ml min^-1^, respectively. Hydrogen was used as carrier gas, at a constant flow rate of 1.0 ml min^-1^. The GC oven program was as follows: initial temperature 100 °C, equilibration time 1.0 min, heat up to 120 °C at the rate of 10 °C min^-1^, hold for 5 min, then heat at the rate of 40 °C min^-1^ until 230 °C and hold for 2 min. Mass spectra were recorded in Selected Ion Monitoring (SIM) mode and following m/z ions were detected at the dwell time of 50 msec: 41, 43, 45, 57, 60, 73, 74, 84. The detector was switched off during the 1 min of solvent delay time. The transfer line, ion source and quadrupole temperatures were set to 230, 230 and 150 °C, respectively. The mass spectrometer was tuned according to manufacturer’s recommendation using perfluorotributylamine (PFTBA). Dilution series of SCFA mixtures were prepared at the concentrations of 1.000, 0.500, 0.250, 0.125, 0.060 and 0.030 mM of each SCFA includind acetic, propionic, butyric, isobutyric, 2-methyl isobutyric, valeric and isovaleric acids (all purchased from Sigma-Aldrich). The data generated from dilution series was used to construct a calibration curve for the absolute quantification. Initial inspection of the GC-MS data was performed using MSD ChemStation software (ver. E.02.02.1431, Agilent Technologies, Inc., Germany). Mass spectra of SCFA were compared against the NIST11 library. SCFA peak areas were integrated from SIM chromatograms using an in-house script in Matlab (version R2015a, The MathWorks, Inc., Massachusetts, USA). Two SCFA, 2-methyl isobutyric acid and isovaleric acid, co-eluted at the retention time range of 4.22-4.45 min, thus peak areas were calculated by deconvoluting these peaks using *m/z* ions 74 for 2-methyl isobutyric acid and 60 for isovaleric acid.

**Data Analysis**

P values for all 331 variables of the combined dataset were calculated for gender and treatment effects based on one way ANOVA using *anova1* function. For the treatment effect multiple comparison test was also performed to determine which pairs of group means (treatment types) are significantly different. This was done using *multcompare* function. All P values were corrected for the false discovery rate (FDR) for multiple hypothesis testing using mafdr function. anova1 and multcompare functions were available through a stats toolbox and *mafdr* was available through *bioinfo* toolbox of the Matlab version R2015a (8.5.0.197613).

ASCA partitioned data variation according to the study design factors including, treatment, gender and individual effects and their two-factor interaction effects as the overall variance contribution of effects across all metabolites. The null hypothesis (H0) was checked for each main and interaction effect using sum of squares of effect matrices, e.g. XDAF, and p-values were assessed by permutation test (5,000 permutations).

**Fig. S1** Scores plots of the PCA models calculated on X1 (a, b and c), ∆X(d, e and f) and on the concatenated X0 and X1 (XT0;XT1) (g, h and i). Scores are colored by individual (a, d and g), gender (b, e and h) or treatment (c, f and i)


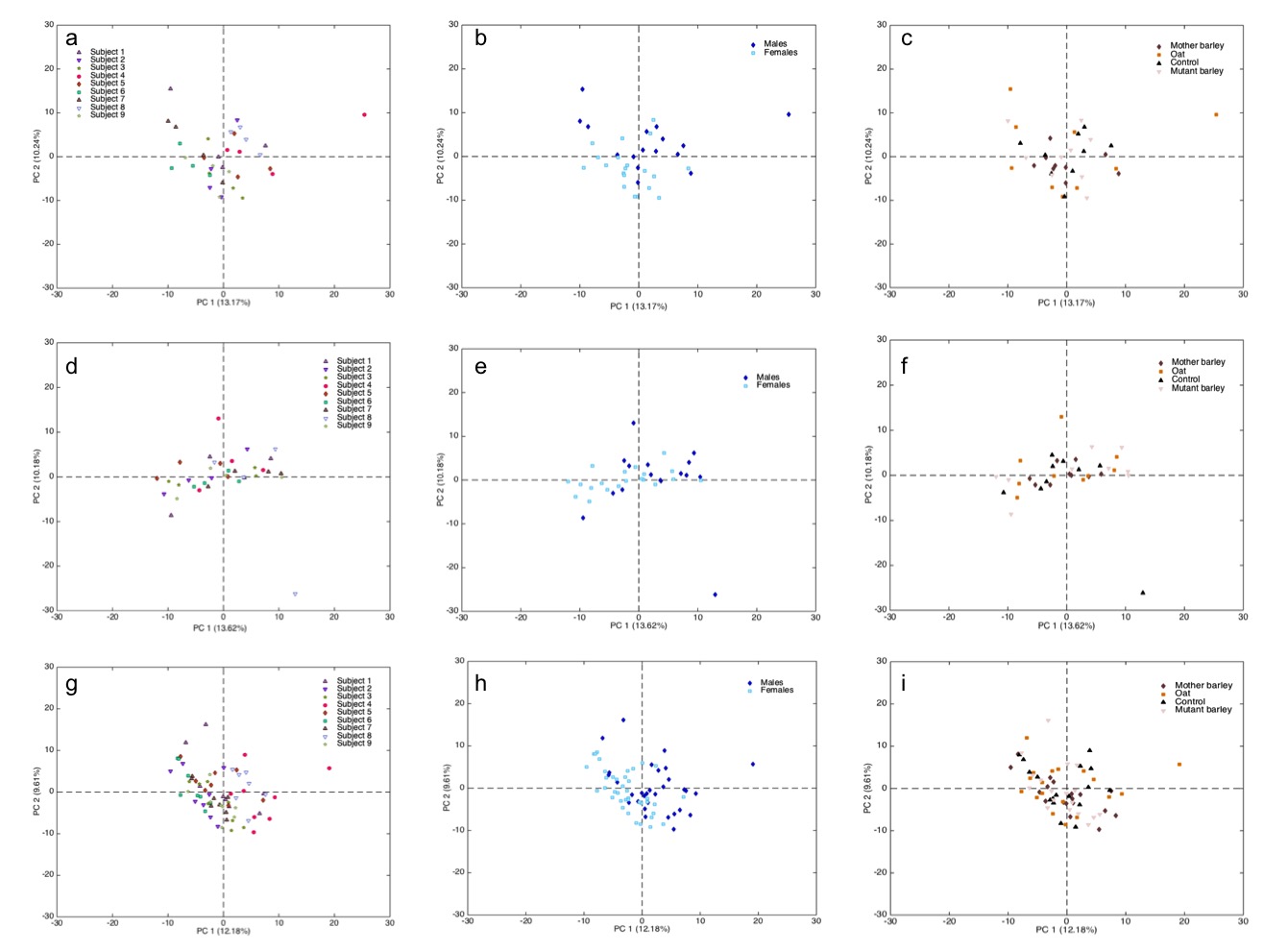


**Table S1** List of peaks found by GC-TOF-MS in fecal samples, with the retention index (RI) found and reported. 102 peaks could not be assigned (“unk”). Metabolites are either level 2 (putatively annotated compounds), when identified, level 3 (Putatively characterised compound classes) or level 4 (Unknown compounds) according to the MSI (Sumner et al. 2007). In the last column, it is also reported which metabolites are markers for a specific treatment and gender.

| № | Metabolite Name | RI found | RI reported | EI-MS match | Marker for |
| --- | --- | --- | --- | --- | --- |
| 1 | unk1 | 940 |  |  |  |
| 2 | Isopropylbenzene | 951 | 969.08 | 898 |  |
| 3 | Ethanimidic acid-2TMS | 960 | 905e | 886 |  |
| 4 | Hexamethyldisilthiane | 965 | 993 | 871 | Control Females |
| 5 | Formamide-2TMS | 968 | 775e | 897 |  |
| 6 | Carbodiimide-2TMS | 971 |  | 878 |  |
| 7 | 4,5-Dimethyl-1-hexene | 975 | 747.4e | 857 |  |
| 8 | Valeric acid-1TMS | 979 | 975 | 954 |  |
| 9 | unk2 | 982 |  |  |  |
| 10 | (Z)-Bis-1,2-(trimethylsilyloxy)ethylene | 987 | 796e | 899 |  |
| 11 | Ethylene glycol-2TMS | 990 | 993 | 892 | MLBG Females |
| 12 | 1,2-Propanediol-2TMS | 994 | 1013 | 840 | Control Males |
| 13 | unk3 | 1002 |  |  |  |
| 14 | unk4 | 1029.41 |  |  |  |
| 15 | unk5 |  |  |  |  |
| 16 | Ethanolamine-2TMS | 1114.01 | 1020.08 | 881 |  |
| 17 | reagent peak | 1034 |  |  |  |
| 18 | Glycine-1TMS | 1081.35 | 1105 | 800 |  |
| 19 | 1,3 Propanediol-2TMS | 1130.29 | 1050.05 | 853 |  |
| 20 | Lactic acid-2TMS | 1056.887 | 1057 | 951 |  |
| 21 | unk6 | 1135.58 |  |  |  |
| 22 | Mercaptoacetic acid-2TMS | 1184.52 | 1055 | 756 |  |
| 23 | Caproic acid-2TMS | 1190.22 | 1071 | 956 |  |
| 24 | Glycolic acid-2TMS | 1214.49 | 1072 | 899 | Barley Females |
| 25 | Alanine-2TMS | 1114.31 | 1105 | 901 |  |
| 26 | Glycine-2TMS | 1223.18 | 1105 |  |  |
| 27 | β-Hydroxypyruvic acid-2TMS | 1204.21 | 1115 | 755 |  |
| 28 | Pyridine, 3-trimethylsiloxy- | 1185.23 | 1134.01 | 819 |  |
| 29 | β-Lactic acid-2TMS | 1190.53 | 1141 | 892 |  |
| 30 | 3-hydroxybutanoic acid-2TMS | 1177.24 | 1147.09 | 904 | Barley Females |
| 31 | Butanoic acid, 2-hydroxy-3-methyl-2TMS | 1226.19 | 1172 | 893 |  |
| 32 | d-2-Aminobutyric acid2-TMS | 1256.15 | 1161.07 | 927 | MLBG Males |
| 33 | unk7 | 1261.44 |  |  | Barley Females |
| 34 | β-Amino isobutyric acid-2TMS | 1223.49 | 1137e | 887 |  |
| 35 | Cyclohexanecarboxylic acid-1TMS | 1278.13 | 1188 | 921 |  |
| 36 | unk8 | 1352.29 |  |  |  |
| 37 | Valine-2TMS | 1256.45 | 1234 | 936 |  |
| 38 | unk9 | 1300.53 |  |  |  |
| 39 | unk10 | 1249.17 |  |  |  |
| 40 | 2-Hydroxyisocaproic acid-2TMS | 1319.14 | 1241 | 870 |  |
| 41 | Norvaline-2TMS | 1328.24 | 1236 | 866 |  |
| 42 | Urea-2TMS | 1337.34 | 1243 | 802 | Barley Males |
| 43 | Benzoic acid-1TMS | 1243.987 | 1232 | 938 |  |
| 44 | unk11 | 1346.44 |  |  |  |
| 45 | unk12 | 1320.56 |  |  |  |
| 46 | Ethyl 1-methylpipecolinate | 1256.025 | 1260 | 918 |  |
| 47 | unk13 | 1400.03 |  |  |  |
| 48 | Octanoic acid-1TMS | 1269.21 | 1260 | 939 |  |
| 49 | Leucine-2TMS | 1278.31 | 1272 | 896 |  |
| 50 | Glycerol-3TMS | 1383.25 | 1292 | 905 |  |
| 51 | Isoleucine-2TMS | 1436.43 | 1306 | 882 |  |
| 52 | Isoleucine-2TMS | 1375.57 | 1306 | 905 |  |
| 53 | Nicotinic acid-1TMS | 1315.12 | 1308 | 917 |  |
| 54 | Proline-2TMS | 1315.12 | 1297.39 | 864 |  |
| 55 | Phenylacetic acid1TMS | 1420.06 | 1279 | 930 |  |
| 56 | reagent peak | 1299.362 |  |  |  |
| 57 | unk14 |  |  |  |  |
| 58 | unk15 | 1333.32 |  |  |  |
| 59 | unk16 |  |  |  | Oat Males |
| 60 | Succinic acid-2TMS | 1311.04 | 1314 | 937 |  |
| 61 | unk17 | 1421.48 |  |  |  |
| 62 | unk18 | 1456.47 |  |  |  |
| 63 | unk19 | 1430.59 |  |  |  |
| 64 | Glyceric acid-3TMS | 1430.59 | 1342 | 879 |  |
| 65 | Oxamide-2TMS | 1335.15 |  | 848 |  |
| 66 | 5-hydroxy-n-valeric acid-2TMS | 1405.11 | 1314 | 905 |  |
| 67 | Uracil-2TMS | 1344.25 | 1330 | 909 |  |
| 68 | unk20 | 1449.19 |  |  |  |
| 69 | unk21 | 1353.35 |  |  |  |
| 70 | Fumaric acid-2TMS | 1432.41 | 1345 | 894 |  |
| 71 | unk22 | 1467.39 |  |  |  |
| 72 | Serine-3TMS | 1354.737 | 1342.06 | 887 |  |
| 73 | Nonylic acid-1TMS | 1381.06 | 1358 | 847 |  |
| 74 | unk23 | 1451.02 |  |  |  |
| 75 | unk24 | 1520.58 |  |  |  |
| 76 | unk25 | 1399.26 |  |  |  |
| 77 | Threonine-3TMS | 1378.813 | 1367.04 | 936 |  |
| 78 | art | 1452.45 |  |  |  |
| 79 | unk26 | 1426.57 |  |  |  |
| 80 | unk27 | 1461.55 |  |  |  |
| 81 | Thymine-2TMS | 1401.09 | 1400 | 848 |  |
| 82 | unk28 | 1436.07 |  |  |  |
| 83 | unk29 | 1506.03 |  |  |  |
| 84 | unk30 | 1506.03 |  |  |  |
| 85 | Glutaric acid -2TMS | 1445.17 | 1400 | 885 |  |
| 86 | Methionine-1TMS | 1514.07 | 1411.04 | 873 |  |
| 87 | Hydrocinnamic acid-1TMS | 1559.35 | 1397 | 896 |  |
| 88 | unk31 | 1462.08 |  |  |  |
| 89 | unk32 | 1430.706 |  |  |  |
| 90 | unk33 | 1444.48 |  |  |  |
| 91 | unk34 | 1437.56 |  |  |  |
| 92 | unk35 | 1535.44 |  |  |  |
| 93 | Decanoic acid-1TMS | 1455.36 | 1455 | 957 |  |
| 94 | 2-Deoxy ribose-3TMS | 1541.08 | 1431 | 789 |  |
| 95 | reagent peak | 1563.52 |  |  |  |
| 96 | Aspartic acid-3TMS | 1563.52 | 1502.04 | 767 |  |
| 97 | 1-Mono-isobutyrin-2TMS | 1523.48 | 1324e | 901 |  |
| 98 | unk36 | 1551.56 |  |  |  |
| 99 | unk37 | 1637.28 |  |  | Barley Females |
| 100 | Deoxyribopyranose-3TMS | 1597.24 | 1502 | 893 |  |
| 101 | unk38 | 1620.09 |  |  |  |
| 102 | Adipic acid-2TMS | 1562.45 | 1498 | 795 |  |
| 103 | 2-Deoxy ribose-3TMS | 1625.33 | 1502.04 | 888 |  |
| 104 | trans-4-Trimethylsilyloxy-cyclohexyl(trimethylsilyl)carboxylate | 1509.24 | 1511 | 858 |  |
| 105 | Pyroglutamic acid-2TMS | 1630.57 | 1520.07 | 915 |  |
| 106 | Glutaminic acid-2TMS | 1596.17 | 1511 | 803 |  |
| 107 | Butanoic acid, 2,3,4-trihydroxy-3TMS | 1538.02 | 1523 | 871 |  |
| 108 | reagent peak | 1561.37 |  |  |  |
| 109 | Phenylalanine-1TMS | 1561.37 | 1554 | 880 |  |
| 110 | unk39 | 1607.05 |  |  |  |
| 111 | Butanoic acid, 2,3,4-trihydroxy-3TMS | 1629.49 | 1523 | 887 |  |
| 112 | reagent peak | 1652.33 |  |  |  |
| 113 | Benzoic acid, 3-hydroxy-2TMS | 1612.29 | 1561 | 765 |  |
| 114 | unk40 | 1635.14 |  |  |  |
| 115 | unk41 | 1569.157 |  |  |  |
| 116 | 2-Hydroxyglutaric acid-3TMS | 1572.29 | 1580 | 912 |  |
| 117 | unk42 | 1640.38 |  |  |  |
| 118 | unk43 | 1623.18 |  |  |  |
| 119 | Pentanoic acid, 2,2,4-trimethyl-3-carboxyisopropyl, isobutyl ester | 1748.54 | 1605 | 882 |  |
| 120 | reagent peak | 1589.719 |  |  |  |
| 121 | unk44 | 1628.42 |  |  |  |
| 122 | 3-Hydroxyphenylacetic acid-2TMS | 1603.674 | 1600 | 926 |  |
| 123 | D-(-)-Ribofuranose, tetrakis(trimethylsilyl) ether (isomer 1) | 1628.57 | 1623 | 798 |  |
| 124 | DL-Arabinopyranose-4TMS | 1621.49 | 1607 | 921 |  |
| 125 | 4-Trimethylsilyloxycyclohexylacetate, trimethylsilyl ester | 1614.41 | 1501e | 844 |  |
| 126 | Phenylalanine-2TMS | 1758.58 | 1623 | 889 |  |
| 127 | L-(+)-Rhamnopyranose-4TMS | 1758.58 | 1618 | 891 |  |
| 128 | Nitrilotris(ethyleneoxy)-3TMS | 1622.779 | 1645 | 883 |  |
| 129 | reagent peak | 1744.43 |  |  |  |
| 130 | D-(-)-Ribofuranose-4TMS (isomer 1) | 1658.19 | 1623.05 | 860 |  |
| 131 | D-(-)-Ribofuranose-4TMS (isomer 2) | 1644.04 | 1641.01 | 852 |  |
| 132 | p-Hydroxyphenylacetic acid-2TMS | 1634.536 | 1627 | 910 |  |
| 133 | D-Arabinopyranose-4TMS (isomer 2) | 1795.28 | 1636.05 | 912 |  |
| 134 | unk45 | 1641.884 |  |  |  |
| 135 | D-(-)-Ribofuranose-4TMS (isomer 2) | 1701.57 | 1641 | 789 |  |
| 136 | DL-Arabinopyranose-4TMS | 1694.49 | 1643.04 | 912 |  |
| 137 | unk46 | 1687.41 |  |  |  |
| 138 | Lauric acid-1TMS | 1766.57 | 1651 | 945 |  |
| 139 | α-L-(-)-Fucopyranose-4TMS | 1824.51 | 1658.06 | 851 |  |
| 140 | D-(-)-Ribofuranose-4TMS (isomer 1) | 1724.11 | 1671 | 835 |  |
| 141 | unk47 | 1672.746 |  |  |  |
| 142 | unk48 | 1782.04 |  |  |  |
| 143 | Mannose, 6-deoxy-2,3,4,5-tetrakis-O-(trimethylsilyl)-, L- | 1767.49 | 1686 | 893 | Control Females |
| 144 | unk49 | 1767.49 |  |  |  |
| 145 | reagent peak | 1739.18 |  |  |  |
| 146 | β-L-(-)-Fucopyranose-4TMS | 1732.11 | 1697.04 | 849 |  |
| 147 | α-D-Xylopyranose-4TMS | 1717.55 | 1703 | 896 |  |
| 148 | 1,2,5,6-Hexanetetrol-4TMS | 1710.956 | 1626e | 835 |  |
| 149 | unk50 | 1847.57 |  |  |  |
| 150 | 3-Hydroxyphenylpropionic acid-2TMS | 1840.49 | 1717 | 932 |  |
| 151 | unk51 | 1819.26 |  |  |  |
| 152 | unk52 | 1805.11 |  |  |  |
| 153 | unk53 | 1790.55 |  |  |  |
| 154 | unk54 | 1870.11 |  |  |  |
| 155 | β-D-(+)-Xylopyranose-4TMS | 1848.48 | 1754.08 | 898 |  |
| 156 | unk55 | 1834.33 |  |  |  |
| 157 | unk56 | 1820.18 |  |  |  |
| 158 | reagent peak | 1798.55 |  |  |  |
| 159 | unk57 | 1798.55 |  |  |  |
| 160 | Azelaic acid-2TMS | 1928.56 | 1787 | 863 |  |
| 161 | D-(-)-Tagatofuranose-5TMS (isomer 1) | 1921.48 | 1800.06 | 778 |  |
| 162 | α-D-Mannopyranose-5TMS | 1835.25 | 1890.03 | 917 |  |
| 163 | D-(-)-Tagatofuranose-5TMS (isomer 1) | 1821.09 | 1800.06 | 854 |  |
| 164 | D-(-)-Fructopyranose-5TMS (isomer 1) | 1860.08 | 1802.04 | 865 |  |
| 165 | Tetradecanoic acid-1TMS | 1806.27 | 1788 | 890 |  |
| 166 | D-(+)-Talofuranose-5TMS (isomer 1) | 1864.43 | 1822.05 | 844 |  |
| 167 | unk58 | 1873.53 |  |  |  |
| 168 | 3,4-Dihydroxyphenylacetic acid-3TMS | 1873.53 | 1832 | 940 |  |
| 169 | unk59 (sugar) | 1878.28 |  |  | Oat Males |
| 170 | unk60 (sugar) | 1829.22 |  |  |  |
| 171 | D-(+)-Talofuranose-5TMS(isomer 2) | 1999.37 | 1840.08 | 770 |  |
| 172 | Tetradecanoic acid | 2008.47 | 1841.12 | 925 |  |
| 173 | D-(+)-Galactopyranose-5TMS (isomer 1) | 1901.23 | 1846.07 | 889 |  |
| 174 | Sorbose-5TMS | 1850.658 | 1866.06 | 796 |  |
| 175 | Xylose-MEOX-4TMS | 1905.58 | 1853 | 710 |  |
| 176 | Pentadecyl-1TMS | 1910.33 | 1866 | 838 |  |
| 177 | unk61 | 1973.25 |  |  |  |
| 178 | unk62 (sugar) | 1924.18 |  |  |  |
| 179 | Tyrosine-2TMS | 2040.52 | 1901 | 889 |  |
| 180 | D-(+)-Galactopyranose-5TMS (isomer 2) | 1942.38 | 1889.01 | 899 |  |
| 181 | n-Pentadecanoic acid-1TMS | 2010.05 | 1942 | 888 |  |
| 182 | n-Pentadecanoic acid-1TMS | 2072.57 | 1942 | 871 |  |
| 183 | D-Sorbitol-6TMS | 1965.34 | 1919.07 | 861 |  |
| 184 | reagent peak | 1920.623 |  |  |  |
| 185 | D-Sorbitol-6TMS | 2082.07 | 1919.07 | 861 |  |
| 186 | Tyrosine-3TMS | 1930.618 | 1952 | 917 |  |
| 187 | Hydrocaffeic acid-3TMS | 2037.35 | 1950 | 913 |  |
| 188 | unk63 | 1983.54 |  |  |  |
| 189 | n-Pentadecanoic acid-1TMS | 1988.29 | 1942 | 902 |  |
| 190 | 1-Hexadecanol-1TMS | 2055.55 | 1965 | 874 |  |
| 191 | unk64 | 2118.47 |  |  |  |
| 192 | β-D-Glucopyranose-5TMS | 2015.59 | 2009.05 | 893 |  |
| 193 | unk65 | 2141.42 |  |  |  |
| 194 | unk66 | 2034.19 |  |  |  |
| 195 | Hexadecanoic acid-1TMS | 2052.54 | 2036 | 889 |  |
| 196 | Hexadecanoic acid-1TMS | 2164.54 | 2036 | 933 |  |
| 197 | unk67 | 2030.43 |  |  |  |
| 198 | Palmitoleic acid-1TMS | 2169.33 | 2016 | 874 | BG Males |
| 199 | myo-Inositol-6TMS | 2112.18 | 2152 | 949 |  |
| 200 | N-Acetyl-D-glucosamine-4TMS (isomer 1) | 2170.37 | 2087.05 | 883 |  |
| 201 | Heptadecanoic acid-1TMS | 2233.36 | 2136 | 867 |  |
| 202 | unk68 | 2153.05 |  |  |  |
| 203 | Heptadecanoic acid-1TMS | 2265.05 | 2136 | 837 |  |
| 204 | unk69 | 2157.44 |  |  |  |
| 205 | Caffeic acid-3TMS | 2162.23 | 2141 | 888 |  |
| 206 | Heptadecanoic acid-1TMS | 2167.02 | 2136 | 893 |  |
| 207 | Octadecoxy-1TMS | 2171.41 | 2159 | 856 |  |
| 208 | Linoleic acid ethyl ester | 2256.51 | 2139 | 889 |  |
| 209 | Oleic acid-1TMS | 2166.895 | 2171 | 797 |  |
| 210 | Linoleic acid-1TMS | 2261.771 | 2240 | 927 | Control Males |
| 211 | Linoleic acid-1TMS | 2330.42 | 2240 | 897 | Control Males |
| 212 | reagent peak | 2313.55 |  |  |  |
| 213 | trans-9-Octadecenoic acid-1TMS | 2395.35 | 2215 | 779 |  |
| 214 | unk69 (fatty acid) | 2378.48 |  |  |  |
| 215 | unk70 | 2306.336 |  |  |  |
| 216 | unk71 | 2410.05 |  |  |  |
| 217 | cis-11-eicosenoic acid | 2384.53 | 2356 | 710 |  |
| 218 | 9-Octadecenamide, (Z)- | 2360.589 | 2375 | 870 |  |
| 219 | unk72 | 2539.51 |  |  | Control Females |
| 220 | Dehydroabietic acid-1TMS | 2531.27 | 2385 | 830 |  |
| 221 | Hexanedioic acid, bis(2-ethylhexyl) ester | 2385.778 | 2382 | 921 |  |
| 222 | Myo-Inositol-5TMS, bis(trimethylsilyl) phosphate | 2464.16 |  | 878 | Barley Females |
| 223 | unk73 | 2484.54 |  |  |  |
| 224 | 11-Eicosenoic acid-1TMS | 2542.32 | 2393 | 865 | Control Males |
| 225 | unk74 (fatty acid) | 2472.56 |  |  |  |
| 226 | Eicosanoic acid1-TMS | 2460.58 | 2437 | 893 |  |
| 227 | Inosine-4TMS | 2600.27 | 2596 | 821 |  |
| 228 | 1-Monopalmitin-1TMS | 2588.29 | 2606 | 821 |  |
| 229 | unk75 (fatty acid) | 2716.43 |  |  |  |
| 230 | unk76 (fatty acid) | 2773.26 |  |  |  |
| 231 | Docosanoic acid-1TMS | 2786.52 | 2634 | 847 |  |
| 232 | Maltose-8TMS | 2743.35 | 2649.03 | 807 |  |
| 233 | unk77 | 2748.03 |  |  |  |
| 234 | 4-O-β-Galactopyranosyl-D-mannopyranose, octakis(trimethylsilyl) ether (isomer 2) | 2752.32 | 2624 | 870 | Control Females |
| 235 | unk78 | 2818.12 |  |  |  |
| 236 | Maltose-8TMS | 2831.38 | 2649.03 | 910 |  |
| 237 | unk79 | 2740.36 |  |  |  |
| 238 | 9-Octadecenoic acid, 1,3-bis-(OTMS)-2-propyl ester | 2745.681 | 2746 | 791 | Control Females |
| 239 | 1-Monooleoylglycerol-1TMS | 2806.16 | 2784 | 880 |  |
| 240 | Maltitol | 2810.45 |  | 895 |  |
| 241 | Squalene | 2822.43 | 2809.01 | 903 |  |
| 242 | Enterolactone | 2873.54 | 2889 | 671 | Control Females |
| 243 | Tetracosanoic acid-1TMS | 2899.29 | 2831.06 | 900 | Control Females |
| 244 | unk80 (sugar) | 2989.16 |  |  |  |
| 245 | unk81 | 2938.31 |  |  |  |
| 246 | unk82 | 3105.04 |  |  | Control Females |
| 247 | unk83 | 2990.35 |  |  |  |
| 248 | Tocopherol-γ-tms-derivative | 3067.21 | 3014 | 718 | Control Females |
| 249 | unk84 | 3131.06 |  |  |  |
| 250 | unk85 | 3121.37 |  |  |  |
| 251 | Cerotic acid | 3059.27 | 2962e | 877 |  |
| 252 | Coprostan-3-ol-1TMS | 3209.31 | 3059 | 872 |  |
| 253 | unk86 | 3188.43 |  |  |  |
| 254 | unk87 | 3240.28 |  |  |  |
| 255 | α-Tocopherol,-1TMS | 3214.28 | 3226 | 786 |  |
| 256 | Cholesterol-1TMS | 3261.01 | 3178 | 908 |  |
| 257 | unk88 | 3198.51 |  |  |  |
| 258 | unk89 | 3291.58 |  |  | Control Females |
| 259 | unk90 | 3333.19 |  |  |  |
| 260 | Cholestenone | 3253.02 | 3245 | 830 |  |
| 261 | reagent peak | 3217.47 |  |  |  |
| 262 | Cycloartanyl acetate | 3326.58 | 3356 | 771 |  |
| 263 | Campesterol-1TMS | 3256.28 | 3220.07 | 876 |  |
| 264 | unk91 | 3316.22 |  |  |  |
| 265 | 3-α-Trimethylsilyloxycholanic acid-1TMS | 3376.16 | 3204 | 889 |  |
| 266 | unk92 | 3268.015 |  |  |  |
| 267 | Deoxycholic acid, tris(trimethylsilyl) deriv. | 3330.24 | 3031 | 890 |  |
| 268 | β-Sitosterol-TMS | 3453.37 | 3293.06 | 747 |  |
| 269 | Stigmastanol-1TMS | 3478.16 | 3348 | 815 |  |
| 270 | unk93 | 3502.54 |  |  |  |
| 271 | unk94 | 3409.24 |  |  |  |
| 272 | unk95 | 3447.36 |  |  |  |
| 273 | unk96 | 3491.37 |  |  |  |
| 274 | unk97 | 3507.49 |  |  |  |
| 275 | unk98 | 3485.102 |  |  |  |
| 276 | unk99 | 3694.16 |  |  |  |
| 277 | unk100 | 3743.02 |  |  |  |
| 278 | unk101 | 3731.121 |  |  |  |
| 279 | unk102 | 3981.37 |  |  |  |

**Table S2** Explained variation and P-values of Individual and Treatment main effects evaluated using ASCA, both for ∆X and X1, for the combined data, GC-TOF-MS, SCFA, and metadata

|  | | **ΔX** | | **X_T1_** | |
| --- | --- | --- | --- | --- | --- |
|  |  | P-value | Explained variance (%) | P-value | Explained variance (%) |
| Combined data | Individual | 0.643 | 17.9 | 1.82e-13 | 27.7 |
|  | Treatment | 0.782 | 7.9 | 0.727 | 6.6 |
| GC-TOF-MS | Individual | 0.808 | 17.1 | 5.26e-07 | 23.7 |
|  | Treatment | 0.904 | 7.4 | 0.735 | 7 |
| SCFA | Individual | 0.192 | 26 | 1.49e-10 | 56.2 |
|  | Treatment | 0.0527 | 12.9 | 0.157 | 5.3 |
| Metadata | Individual | 0.109 | 18.3 | 1e-09 | 44.9 |
|  | Treatment | 0.0625 | 10.5 | 0.602 | 4.4 |

**Table S3** Explained variation and P-values of Gender and Treatment main effects as well as their two factor interaction effect evaluated using ASCA, both for ∆X and X1, for the combined data, GC-TOF-MS, SCFA, and metadata

|  | | **ΔX** | | **X_T1_** | |
| --- | --- | --- | --- | --- | --- |
|  |  | P-value | Explained variation (%) | P-value | Explained variation (%) |
| Combined data | Gender | 0.0765 | 4.3 | 0.000199 | 6.9 |
|  | Treatment | 0.799 | 8.8 | 0.991 | 7.1 |
|  | Gender x Treatment | 0.874 | 8.6 | 0.479 | 9.6 |
| GC-TOF-MS | Gender | 0.134 | 4.2 | 0.0255 | 5.2 |
|  | Treatment | 0.908 | 8.3 | 0.96 | 7.6 |
|  | Gender x Treatment | 0.78 | 8.9 | 0.246 | 10.6 |
| SCFA | Gender | 0.00689 | 12.3 | 0.000237 | 22.1 |
|  | Treatment | 0.16 | 12.4 | 0.932 | 3.7 |
|  | Gender x Treatment | 0.94 | 4 | 0.99 | 2.4 |
| Metadata | Gender | 0.295 | 3.6 | 3.05e-07 | 14.9 |
|  | Treatment | 0.0909 | 11.8 | 0.997 | 4.8 |
|  | Gender x Treatment | 0.888 | 7.6 | 1 | 4.4 |

**Results from Oat vs Control comparison.**

Oat MLBG had a greater impact on males' fecal metabolome. The variable, unknown 16, was able to discriminate this intervention group from control (Fig. 3) and was consistently found to be a marker for oat MLBG using both univariate ANOVA approach and a multivariate PLS-DA (Table 1). Unknown 16 was later also found to be a distinctive marker of oat consumption, since it discriminates the oat MLBG group from the barley MLBG group (see section 3.2.3) which is a good indication of the performance of our variable selection and validation method, but not good for the MLBG relevance.

Two metabolites were mostly responsible for this discrimination among females. Both these variables decreased during the oat MLBG treatment. One of them is enterolactone, a molecule which can be found in human biofluids, especially after the ingestion of berry and berry derived products (Mazur et al. 2000). The amount of enterolactone produced by lignan metabolism depends on the presence of a specific microbiota, which in turn is influenced by the diet (Mazur et al. 2000). Therefore, the diets differing in their MLBG content in this intervention study could affect the metabolism of the lignan, which may be the reason for the reduced level of enterolactone in oat MLBG group.
